# St. Marylebone Health Society

**Published:** 1916-03-11

**Authors:** 


### St. Marylebone Health Society.

### COMBINED MUNICIPAL AND VOLUNTARY WORK.

In view of the rapid increase of schemes for infant welfare, the proceedings of the St. Marylebone Health Society, a pioneer in this branch of public health work, are of considerable interest. At the annual meeting, held at the Royal Society of Medicine on February 29, Dr. Porter, the medical officer of health for Marylebone, stated that the infantile death-rate of the borough had fallen from 122 per thousand in 1906, when the society began its work, to 96 per thousand in 1915, and, in his opinion, this gain of 26 in a thousand infant lives was due in a great measure to the labours of the society. Dr. Eric Pritchard pointed out that the standard of maternal care in the district had been raised astonishingly in the same period. It is a trivial but significant fact that whereas eight years ago long-tube bottles and tight buckram binders were extremely common, now he could not obtain a specimen of these abominations when he wanted one for an exhibition. Dr. Pritchard attributed the success of the society to the fact that they combined both voluntary and municipal workers, the amateur and official elements constituting a useful check on each other. In view of the hot controversies now raging in many quarters on this point, Dr. Pritchard's opinion, based on exceptionally wide experience, is deserving of note.

Another speaker, the Bishop of Willesden, put in a useful plea for the elimination of two of the root causes of infant deaths—bad housing and drink, which are apt to be overlooked at present in our concentration on the personal hygiene of the individual child. He pointed out that the American peoples had abandoned their universal and disgusting habit of expectoration when they realised it was a cause of consumption, and he believed that our people would consent to make similar changes in their national habits if they were properly educated to understand the true causes of infant deaths.

